# The Electrochemical Behavior of Carbon Fiber Microelectrodes Modified with Carbon Nanotubes Using a Two-Step Electroless Plating/Chemical Vapor Deposition Process

**DOI:** 10.3390/s17040725

**Published:** 2017-03-30

**Authors:** Longsheng Lu, Linsheng Liang, Kwok Siong Teh, Yingxi Xie, Zhenping Wan, Yong Tang

**Affiliations:** 1 School of Mechanical & Automotive Engineering, South China University of Technology, 381# Wushan Road, Guangzhou 510641, China; l.linsheng@mail.scut.edu.cn (L.L.); yingxix@gmail.com (Y.X.); zhpwan@scut.edu.cn (Z.W.); ytang@scut.edu.cn (Y.T.); 2 Department of Mechanical Engineering, University of California, Berkeley, CA 94720, USA; kwok.siong@gmail.com; 3 School of Engineering, San Francisco State University, San Francisco, CA 94132, USA

**Keywords:** carbon fiber, microelectrodes, biosensor, carbon nanotubes, cyclic voltammetry, reproducibility

## Abstract

Carbon fiber microelectrode (CFME) has been extensively applied in the biosensor and chemical sensor domains. In order to improve the electrochemical activity and sensitivity of the CFME, a new CFME modified with carbon nanotubes (CNTs), denoted as CNTs/CFME, was fabricated and investigated. First, carbon fiber (CF) monofilaments grafted with CNTs (simplified as CNTs/CFs) were fabricated in two key steps: (i) nickel electroless plating, followed by (ii) chemical vapor deposition (CVD). Second, a single CNTs/CF monofilament was selected and encapsulated into a CNTs/CFME with a simple packaging method. The morphologies of as-prepared CNTs/CFs were characterized by scanning electron microscopy. The electrochemical properties of CNTs/CFMEs were measured in potassium ferrocyanide solution (K_4_Fe(CN)_6_), by using a cyclic voltammetry (CV) and a chronoamperometry method. Compared with a bare CFME, a CNTs/CFME showed better CV curves with a higher distinguishable redox peak and response current; the higher the CNT content was, the better the CV curves were. Because the as-grown CNTs significantly enhanced the effective electrode area of CNTs/CFME, the contact area between the electrode and reactant was enlarged, further increasing the electrocatalytic active site density. Furthermore, the modified microelectrode displayed almost the same electrochemical behavior after 104 days, exhibiting remarkable stability and outstanding reproducibility.

## 1. Introduction

Microelectrodes are miniaturized working electrodes with micrometer dimensions that can be made with metallic or non-metallic conductors. Due to its tiny dimension, a microelectrode exhibits several unique electrochemical properties, such as a negligible ohmic drop, high detection sensitivity, high mass transfer rate, and enhanced signal-to-noise ratio [1]. These properties have led to its extensive applications in micro biosensors [2] and chemical analysis sensors [3]. Typical microelectrode materials include platinum, gold, silver, and carbon fiber [1]. Carbon fiber (CF) is an attractive electrode material because of its great physicochemical and electrochemical properties, such as its good electrical and thermal conductivities, adequate corrosion resistance, low density, and elasticity [4]. Since a single CF monofilament is only several microns in diameter, it can be directly used as a CF microelectrode (CFME). Due to their microscale volumes and fast response times, CFMEs have been applied in high performance biosensors for detecting secretory elements such as dopamine (DA), and for monitoring signal generation in a single cell in vivo [5,6]. Rodrigo et al. [7] successfully detected glucose concentrations in rats using a CFME biosensor. CFMEs have also been applied in electrochemical analysis and in the monitoring of environmental pollutants [8], owing to their advantageous properties which include a thin diffusion layer, small IR drop, and high signal-to-noise ratio. Yu et al. [9] presented a copper-modified CFME for the electrochemical determination of nitrate in PM_2.5_ (airborne particulate matter with aerodynamic diameters less than 2.5 μm).

The surface structure of CF—which is the only effective working electrode in a CFME—is a disordered graphite layer with a low specific surface area (SSA) and activity, resulting in weak electronic responses that often elude the detection by conventional instruments [10]. For this reason, CF in a CFME is never used as-is, without modification. In fact, the surface of CF used in a CFME is always modified with techniques such as electrochemical oxidation, or derivatized with enzymes and nanoparticles, to enhance its sensitivity and selectivity toward biochemical molecules. Additionally, these modifications can effectively expand the application of CFME in the fields of analytical chemistry, environmental and health science, fuel cells, and biofuel cells [11].

Carbon nanotube (CNT) is a nanomaterial suitable for the modification of the CFME because it can significantly increase the overall surface area without substantially changing the size of an electrode [12]. CNTs have been found to promote electron-transfer reactions, minimize the fouling of electrode surfaces, and enhance the electrocatalytic activity of the electrodes [13]. Some researchers have tried to modify CF microdisk electrodes with single-walled CNTs via dip-coating [14,15] or electrochemical deposition [16]. They successfully used these modified microelectrodes to detect specific biochemical substances like dopamine. Among the existing methods used to modify the CFME with CNTs, a two-step method combining electroless plating and a chemical vapor deposition (CVD) process shows great promise. Using this method [17], a CF-Ni-CNTs coaxial fiber structure was successfully fabricated on the surface of a CF. Experimental results validated the fact that the SSA and capacitance of CF were significantly enhanced by this structure. Furthermore, the CNTs grafted on the CF surface by the CVD technique distribute more uniformly and make contact with the pristine CF surface more intimately. In addition, it is simple to adjust the thickness and density of a CNT layer by varying the CVD process parameters, such as temperature, catalyst composition, and process gas mixture [18,19]. As such, this two-step method is very compatible and scalable for the mass production of CNTs/CF, potentially enabling the low cost fabrication of CNTs/CFMEs exhibiting high productivity [19]. 

In this work, a new cylindrical CFME modified with multi-walled carbon nanotubes (CNTs), denoted as CNTs/CFME, was fabricated and its properties were characterized. First, numerous carbon fiber (CF) monofilaments grafted with CNTs (simplified as CNTs/CFs) that appeared in a bundle were fabricated using a two-step electroless plating/CVD method. Second, a single CNTs/CF monofilament was selected and encapsulated into a CNTs/CFME using a simple sealing method. The electrochemical properties of the as-prepared CNTs/CFMEs with different coating parameters (content, morphology, specific surface area, etc.) were investigated through cyclic voltammetry and chronoamperometry methods. 

## 2. Materials and Methods

### 2.1. Materials and Reagents

The CFs used throughout this work are T700SC-12K-50C polyacrylonitrile (PAN)-based carbon fibers (Toray Industries, Inc., Tokyo, Japan), in the form of a bundle comprising 12,000 monofilaments (12K tow). For a single continuous CF filament, the average diameter is 7 μm and the average resistivity is 1.6 × 10^−3^ Ω·cm (625 S·cm^−1^ in conductivity).

During electroless plating, the sensitizing solution was prepared by mixing SnCl_2_·2H_2_O (10 g·L^−1^) and HCl (40 ml·L^−1^), and the activating solution was prepared by mixing PdCl_2_ (0.5 g·L^−1^) and HCl (20 ml·L^−1^). The solution used for electrochemical tests was 5.0 mM potassium ferrocyanide (K_4_Fe(CN)_6_) aqueous solution, with 1.0 M KCl aqueous solution added as a supporting electrolyte. The purity of the acetylene and argon gases used in this experiment was 99.999 vol%. All of the other reagents were of analytical reagent grade and were used without further purification. 

### 2.2. Preparation of CNTs/CFMEs

**Figure 1:**
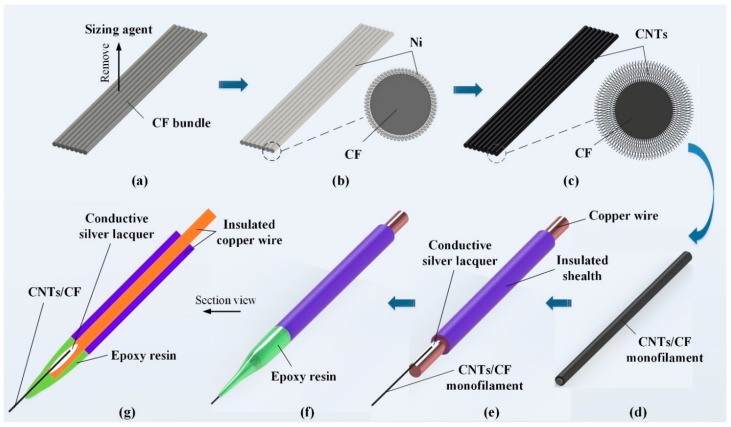
The fabrication process of a CNTs/CFME. (**a**) Pretreatment of the CF bundle surface; (**b**) nickel electroless plating; (**c**) CNTs grafting; (**d**) separating a CF monofilament; (**e**) connecting the CF monofilament to an insulated copper wire; (**f**) encapsulation with epoxy resin; (**g**) section view of the CNTs/CFME structure.

A segment of CFs which was 100 mm-long was tailored from the CF bundle. Then, it was integrally grafted with CNTs. Following this, a single CNTs/CF monofilament was separated and used to make a CNTs/CFME, as shown in Figure 1.

The CNTs were directly grown on the CFs segment that was first coated with catalytic electroless nickel (Ni), followed by a CVD process in an acetylene/argon environment. A series of pre-treatments for CFs were carried out to ensure the successful growth of CNTs [17]. These steps included: (1) removal of the sizing agent on CFs through immersing the samples in acetone for 40 min; (2) sensitization and activation treatments using the aforementioned sensitizing and activating solutions for 10 min sequentially, with ultrasonic vibration assistance; (3) electroless plating of a Ni-layer on the CF surface using a recipe listed in Table 1. To differentiate this work from previous work [17], the plating time was set at 2 min, in order to obtain a thin Ni layer; and (4) growth of the CNTs via a vapor-liquid-solid mechanism via the CVD method in a vacuum tube furnace (FWL(ZK)-08/70/3, Facerom, Hefei, China). During the CVD process, the growth temperature was set to 680 °C, and acetylene gas (20 sccm) was introduced as the carbon source and argon (50 sccm) as the carrier gas. In order to elucidate the influence of CNT dimensions and morphologies on the electrochemical properties of CFMEs, the CNTs growth time was individually controlled to be 5 min, 10 min, and 15 min, to obtain a series of CNTs/CF samples with different CNTs contents and morphologies, which were denoted as CNTs/CF-T5, CNTs/CF-T10, and CNTs/CF-T15, respectively.

After grafting CNTs on CFs, a simple sealing method adopted epoxy resin (E44-6101) to encapsulate CNTs/CFMEs. First, a 10-mm long single CNTs/CF monofilament was selected from the as-prepared CNTs/CF bundle. Then, one end of the monofilament was attached to a piece of insulated copper wire (UL1007 24AWG), which was about 1.43 mm in diameter and 30 mm in length, using conductive silver lacquer (MCN-DJ002, Mechanic, Hongkong, China). In order to minimize contact resistance, the CNTs/CF monofilament overlapped with the exposed copper wire for about 3–4 mm. After the conductive silver lacquer was cured, the junction and adjacent bare part of the copper wire were encapsulated with epoxy resin. Finally, the protruding end of the CNTs/CF was carefully trimmed to 250–300 μm in length under a microscope (VHX-2000, KEYENCE, Osaka, Japan), and a CNTs/CFME was fabricated. As such, a series of CNTs/CFMEs, made from pristine CF, CNTs/CF-T5, CNTs/CF-T10, and CNTs/CF-T15 were prepared, denoted as CFME-T0, CNTs/CFME-T5, CNTs/CFME-T10, and CNTs/CFME-T15, respectively.

### 2.3. Performance Characterization

The surface morphologies of CFs with and without CNTs were observed using a high resolution thermal field emission scanning electron microscope (ZEISS Merlin, Jena, Germany). The modified CNTs were observed using a field emission transmission electron microscope (TEM, JEM-2100F, JEOL Ltd., Tokyo, Japan). The electrical conductivities of CFMEs were expressed using the corresponding CF or CNTs/CFs monofilament, instead of being measured directly, to avoid damage to the electrodes. A four-point probe method [20] was employed to measure the electrical conductivity of single CF or CNTs/CF monofilaments.

**Figure 2:**
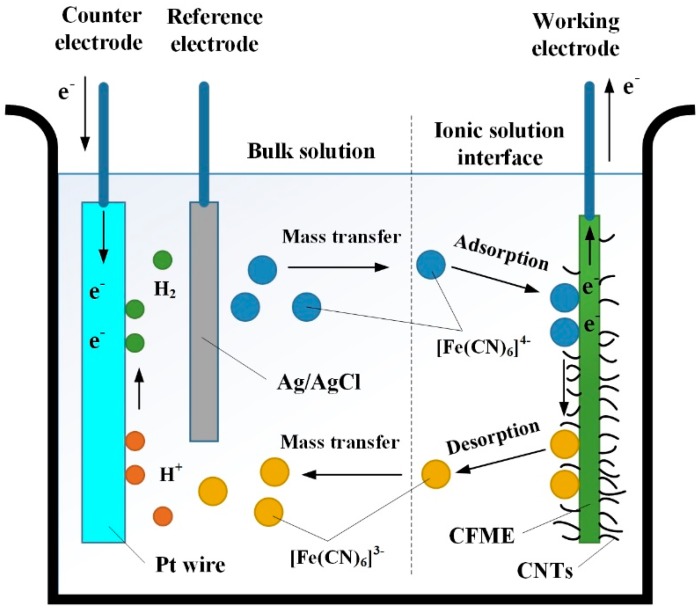
Schematic diagram of the electrochemical reaction under a three-electrode system in K_4_Fe(CN)_6_ aqueous solution.

The SSA of CFs with and without CNTs was measured by N_2_ adsorption tests in a Brunauer-Emmett-Teller (BET) analyzer (ASAP 2020 V4.00, Micromeritics Instrument Corporation, Atlanta, GA, USA). The electrochemical properties of the as-prepared CFME and CNTs/CFME were mainly characterized by cyclic voltammetry, using an electrochemical workstation (CHI 650D, CH Instruments, Shanghai, China) and a three-electrode system, as shown in Figure 2. In the three-electrode system, the working electrode is an as-prepared CFME or CNTs/CFME, the reference electrode is Ag/AgCl (ceramic core, 3.0 M KCl, Φ12 × 120), and the counter electrode is a Pt wire (1 mm in diameter and 37 mm in length). Before electrochemical property tests, both the CFME and CNTs/CFMEs were electrochemically pretreated in 0.5 M H_2_SO_4_ with the cyclic voltammetry method, within a potential range of 0 to +1.0 V at a scan rate of 0.1 V·s^−1^, until a stable cyclic voltammogram was obtained.

## 3. Results and Discussion

### 3.1. Morphology Observation of CNTs/CFs

**Figure 3:**
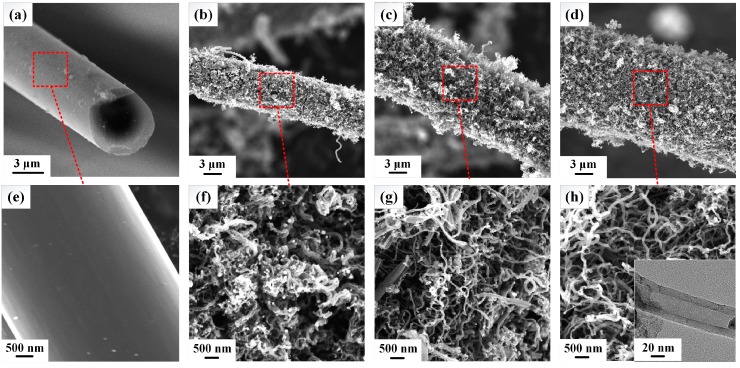
SEM images of the pristine CF and CNTs/CF samples with different lengths of CNTs at different growth times. (**a**) Pristine CF; (**b**) CNTs/CF-T5 (5 min of CNT growth); (**c**) CNTs/CF-T10 (10 min of CNT growth); (**d**) CNTs/CF-T15 (15 min of CNT growth). (**e**–**h**) Magnified images of the surfaces of (**a**–**d**), respectively. The inset in (**h**) shows the TEM image of a CNT grown on the surface of a CF by the CVD method.

Using the aforementioned two-step method, a layer of coaxial CNTs was successfully grown on the CF surface. As shown in Figure 3a–d, the entire surface of the CF is fully covered by slender CNTs, and the diameter of the CNTs/CF composite increases with increasing CNTs growth time. The inset in Figure 3h shows a TEM image of a CNT, clearly indicating that the CNT has a hollow tubular structure like bamboo. The main constituent of a CNT is the carbon element, generated from the decomposition of acetylene on the surface of Ni nanoparticles. Sengupta and Jacob [21] concluded that the dissolved carbon element would diffuse toward the bottom of the Ni particles and segregates as graphite on the CF surface. The Raman spectrum proved that the structure of the CNTs was multi-walled [17]. As the CNTs growth time increases, carbon generated from the decomposition of acetylene increases, causing CNTs to grow in length (as shown in Figure 3f–h), thus enlarging the thickness of the CNT layer, as revealed by Brukh and Mitra [22]. Consequently, the diameter of a CNTs/CF is larger than that of the pristine CF, and increases with increasing CNTs growth time. As can be seen from Figure 3e–h, the surface of the pristine CF is very smooth, but those of CNTs/CF samples are extremely rough and porous, indicating that a CNTs/CF has a larger SSA than a pristine CF. Since the SSA of a CNT (theoretical surface areas for multi-walled CNTs are diameter-dependent and estimated to be in the range of a few hundred m^2^·g^−1^ [23]) is much higher than a pristine CF (0.15 m^2^·g^−1^ obtained from BET analyses), the thicker the CNTs layer, the larger the SSA of a CNTs/CF. Therefore, the SSAs of CNTs/CF-T5, CNTs/CF-T10, and CNTs/CF-T15 were increased remarkably from 0.15 m^2^·g^−1^ to 35.95, 84.97, and 119.53 m^2^·g^−1^, respectively, demonstrating that the SSA of a CF will be significantly enlarged after its surface is covered with CNTs.

**Figure 4:**
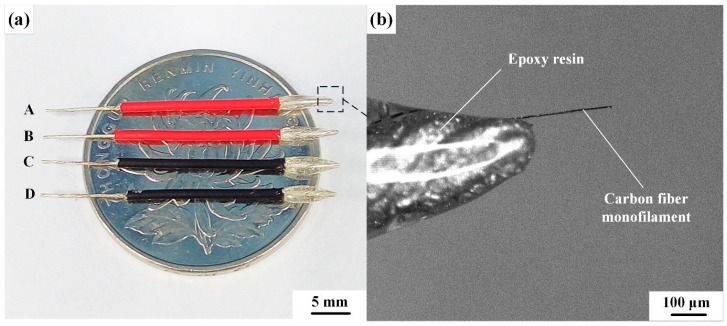
(**a**) Picture of the actual microelectrodes fabricated. A–D is CFME-T0, CNTs/CFME-T5, CNTs/CFME-T10, and CNTs/CFME-T15, respectively; (**b**) Magnified image of the protruding electrode.

The as-fabricated CNTs/CFMEs are shown in Figure 4a. Each CNTs/CFME has two exposed ends. One exposed end is a piece of copper wire, whose length (10 mm in this work) is selected based on the connection requirements to the outer circuits. The other exposed end is a piece of protruding CF monofilament (250–300 μm) modified with or without CNTs, which acts as the working electrode. Figure 4b shows a magnified view of the protruding CF monofilament, which clearly shows that the CF was well encapsulated by epoxy resin. Following proper sample preparation and encapsulation protocols, the epoxy resin keeps the protruding CF clean and away from contaminants. 

### 3.2. Electrical Conductivity Analysis of CNTs/CFs

Our test results show that the electrical conductivities of CNTs/CF-T5, CNTs/CF-T10, and CNTs/CF-T15 were enhanced by 15%, 38%, and 57%, respectively, compared to that of the pristine CF (about 625 S·cm^−1^). This validates the beneficial role of CNTs: that longer, denser CNTs lead to better electrical conductivity in the CNTs/CF. Among the CF and CNTs/CFs, CNTs/CF-T15 has the largest electrical conductivity, of 965.94 S·cm^−1^, which can be attributed to the following two reasons. First, the highly conductive multi-walled CNTs (about 1000–2000 S·cm^−1^ [24]) significantly elevated the conductivity of a pristine CF. Second, and importantly, CNTs with a large length-to-diameter ratio would entangle with each other on the CF surface and form a three-dimensional (3D) coaxial conductive network, providing a larger contact area and additional electron conduction pathways, and thus enhancing the electrical conductivity [25]. By extension, the microelectrodes fabricated from the CNTs/CFs monofilament would therefore have markedly improved electrical conductivity compared to the one made from the pristine CF. 

### 3.3. Cyclic Voltammetry (CV) Analysis of CNTs/CFMEs

**Figure 5:**
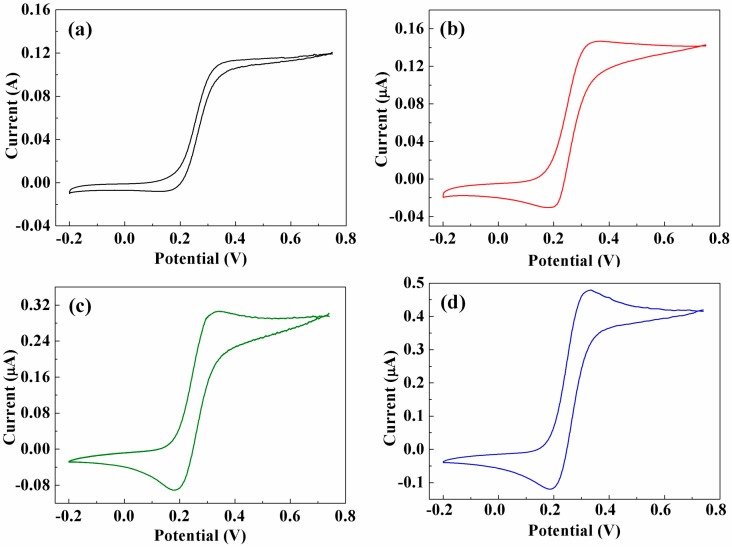
CV curves of (**a**) CFME-T0, (**b**) CNTs/CFME-T5, (**c**) CNTs/CFME-T10 and (**d**) CNTs/CFME-T15 in a 5.0 mM K_4_Fe(CN)_6_ (in 1.0 M KCl) at a scan rate of 0.10 V·s^−1^.

Figure 5 shows the CV curves of the as-prepared CFMEs in 5.0 mM K_4_Fe(CN)_6_, performed at a scan rate of 0.10 V·s^−1^. As shown, all of the CV curves exhibit highly symmetrical shapes for both the forward and reverse potential scans, indicating that highly reversible redox reactions are taking place at the CNTs/CFMEs. It demonstrates that the CNTs/CFMEs, as microelectrodes, exhibit an outstanding electrochemical property in the presence of K_4_Fe(CN)_6_ and are able to reproduce the electrode reaction process of active substances [26]. From Figure 5a, it can be seen that the CV curve of the pure CFME has relatively gentle peaks, low peak currents, and a narrow area under the curve, similarly illustrated by Chen et al. [27]. In comparison, the CNTs/CFMEs show better CV behaviors, e.g., more distinguishable peaks, as well as much higher response currents, as shown in Figure 5b–d. In addition, the microelectrode decorated with longer and denser CNTs has more evident peaks and higher peak currents, demonstrating that the CNTs/CFME has a higher sensitivity than the unmodified CFME [28].

**Figure 6:**
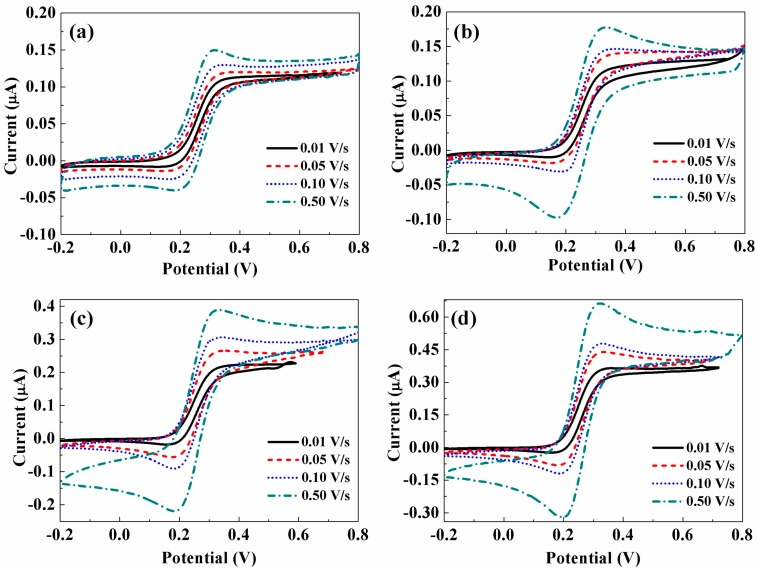
Cyclic voltammograms of (**a**) CFME-T0, (**b**) CNTs/CFME-T5, (**c**) CNTs/CFME-T10, (**d**) CNTs/CFME-T15 in 5.0 mM K_4_Fe(CN)_6_ (in 1.0 M KCl) at a scan rate of 0.01 V·s^−1^, 0.05 V·s^−1^, 0.10 V·s^−1^ and 0.50 V·s^−1^, respectively.

Figure 6 shows the individual CV curves of pure CFME and CNTs/CFMEs at scan rates of 0.01 V·s^−1^, 0.05 V·s^−1^, 0.10 V·s^−1^, and 0.50 V·s^−1^ in 5.0 mM K_4_Fe(CN)_6_. As shown, the peak potentials (including the oxidation peak, *E_pa_*, and reduction peak, *E_pc_*) of identical electrodes at different scan rates show a negligible difference, with *E_pa_* approaching 0.35 V and *E_pc_* displaying a value of 0.17 V for all four electrode samples. 

According to the Nernst equation, the boundary condition of the reversible process can be expressed by [29]:

(1) $$\Delta E_{p}=E_{pa}-E_{pc}\leq \frac{2.3RT}{n\;F}$$

where $\Delta E_{p}$ is the peak-to-peak potential difference, *R* is the universal gas constant (8.3143 J·K^−1^·mol^−1^), *T* is the thermodynamic temperature (298.15 K for room temperature), *n* is the number of electrons involved in the reaction, and *F* is the Faraday constant (96,485.3383 C·mol^−1^). Consequently, Equation (1) can be simplified as:

(2) $$\Delta E_{p}=E_{pa}-E_{pc}\leq \frac{59.0}{n}\;\mathrm{mV}$$

Since the main electrode reaction in the K_4_Fe(CN)_6_ solution is: [Fe(CN)_6_]^4−^ − e^−^ = [Fe(CN)_6_]^3−^, the number of electrons involved *n* is one. Therefore, the boundary condition is theoretically equal to $\Delta E_{p}\leq 59.0\;\mathrm{mV}$. Often, the experimentally observed $\Delta E_{p}$ values are greater than the theoretical value of 59.0 mV.

**Figure 7:**
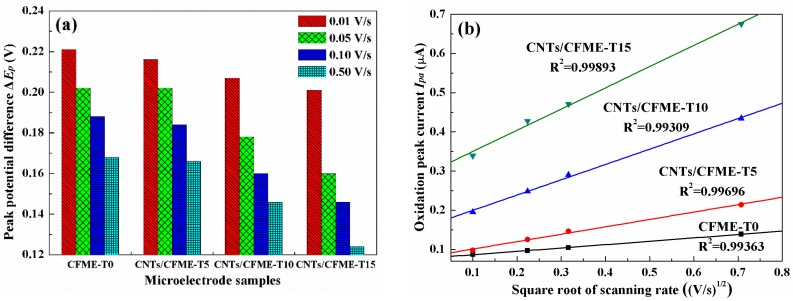
(**a**) $\Delta E_{p}$ values of microelectrodes at different scan rates; (**b**) *I_pa_* values versus the square root of scanning rate.

Figure 7a depicts the $\Delta E_{p}$ values of CFMEs at different scan rates. All experimentally observed $\Delta E_{p}$ values are larger than the theoretical value of 59.0 mV. However, as can be seen from Figure 6, the peak currents *I_p_* for all electrodes increase remarkably with the increasing scan rates of potential, and the ratios of the oxidation peak currents *I_pa_* to the reduction peak currents *I_pc_* approach unity. Therefore, it can be concluded that the reactions occurring at the CNTs/CFMEs and its pristine CFME are reversible or quasi-reversible processes [30]. Figure 7a shows that the $\Delta E_{p}$ values of all CNTs/CFMEs are smaller than that of the pristine CFME at the same potential scan rate. Furthermore, for CNTs/CFMEs, as the CNTs growth time increases—hence producing greater CNT lengths and densities—$\Delta E_{p}$ decreases proportionally toward the theoretical minimum. Such a decrease of $\Delta E_{p}$ implies that the redox reactions taking place at the CNTs/CFMEs tended to be increasingly reversible processes as the dimensions and densities of CNTs increased [31]. 

The oxidation peak current (*I_pa_*) values of all of the electrodes versus the square root of the scan rates (*v*^1/2^) are shown in Figure 7b. The linear relationship between *I_pa_* and *v*^1/2^ is demonstrated with linear fitting, and the coefficients of determination are all over 0.99. Compared to the unmodified CFME, all of the CNTs/CFMEs have increasingly larger linear slopes between *I_pa_* and *v*^1/2^ as the thicknesses of the CNT layers increase, indicating that the peak currents of CNTs/CFMEs have a progressively higher sensitivity towards potential scan rates. As shown in Figure 6, even at a slow scan rate of 0.01 V·s^−1^, the oxidation peaks and reduction peaks of each CNTs/CFME are clearly visible, especially for the CNTs/CFME-T15 electrode. On the contrary, no such peaks were observed for the pristine CFME. This phenomena proves that the CNTs are beneficial to enhancing the sensitivity of CFMEs, and a greater electrode sensitivity to scan rate can be achieved with thicker and denser CNTs [32].

### 3.4. Electrocatalytic Activity of the Microelectrode

**Figure 8:**
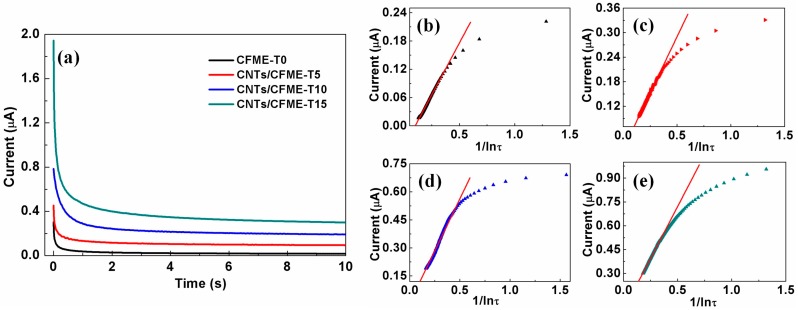
(**a**) The chronoamperogram of the CFME and CNTs/CFMEs in 5.0 mM K_4_Fe(CN)_6_ (in 1.0 M KCl) by applying a step potential of 0.35 V; Plots of chronoamperometry response current vs. l/lnτ of (**b**) CFME-T0; (**c**) CNTs/CFME-T5; (**d**) CNTs/CFME-T10 and (**e**) CNTs/CFME-T15, respectively.

An electrochemical effective area is a critical parameter for characterizing the electrocatalytic activity of an electrode, since it provides the reaction active sites and contact interface area between the electrode and analytes [33]. In light of this, CNTs can appreciably improve the electrochemical performance of a CFME through enlarging its surface area and providing more reaction active sites. The chronoamperogram in Figure 8a shows that the response currents of the CNTs/CFMEs are higher than that of the CFME, which is consistent with the results obtained by the CV method mentioned above. Moreover, the response current decays very slowly in the long time zone, so it can be recognized as a quasi-steady state [34], and its current is defined as the quasi-steady state current *i_qss_*.

Szabo et al. [35] reported an approximate formula describing the relationship between the current and time for cylindrical microelectrodes:

(3) $$i=\frac{nFADc}{r}[\frac{2\exp(-0.05\pi^{\frac{1}{2}}\tau^{\frac{1}{2}})}{\pi^{\frac{1}{2}}\tau^{\frac{1}{2}}}+\frac{1}{\ln(5.2945+0.7493\tau^{\frac{1}{2}})}]$$

where *i* is the current in amps, *A* is the effective electrode area in cm^2^, *D* is the diffusion coefficient in cm^2^·s^−1^, *c* is the concentration of the electroactive species in mol·cm^−3^, *r* is the radius of the cylindrical microelectrode in cm, and *τ* is a coefficient related to time and is equal to $\tau =4Dt/r^{2}$. For a quasi-steady state, since *τ* is very large in the long time zone, Equation (3) can be simplified as [34]:

(4) $$i_{qss}=\frac{2nFADc}{r\ln\tau }$$

Figure 8b–e shows the scatter plots of the chronoamperometry response current vs. l/ln*τ* for different CF electrodes. The response current decays very slowly with the increase of time (after about 0.2 s), confirming the occurrence of the quasi-steady state (the front part of the scatter plots). Therefore, a linear fitting was adopted to fit the plots of this part. Table 2 lists the obtained equations of linear regression, whose coefficients of determination are all larger than 0.99, further validating the good linear relationship between the *i_qss_* and l/ln*τ*. Assuming a diffusion coefficient for 5.0 mM ferrocyanide of 6.67 × 10^−6^ cm^2^·s^−1^ in 1.0 M KCl, as presented by Konopka and Mcduffie [36], the effective electrode area *A* can be calculated according to Equation (4). The results were listed in Table 3. It indicates increased ratios in the effective electrode area of 79%, 443%, and 749% for CNTs/CFME-T5, CNTs/CFME-T10, and CNTs/CFME-T15, respectively, as compared to a pristine CFME. This result is consistent with the SSA variation of CNTs/CFs after the introduction of CNTs, as mentioned above. Thus, the electrocatalytic activities of CNTs/CFMEs are enhanced significantly, owing to the massive active sites provided by the CNTs. 

The chronoamperometry response currents of the electrodes at the tenth second were selected as the quasi-steady state currents and the corresponding current densities were calculated (listed in Table 3). The current densities of the CNTs/CFMEs are much higher than that of an unmodified CFME, with a maximum two times increase (CNTs/CFME-T5). It indicates that the CNTs/CFMEs have a higher mass transfer rate and reaction rate. In addition, the current densities of CNTs/CFME-T10 and CNTs/CFME-T15 are very close, and they are both less than that of CNTs/CFME-T5. It is possible that the diameter of the CF microelectrodes enlarges with the increased content of CNTs, resulting in the reduction of its mass transfer rate.

### 3.5. Reproducibility Analysis of the CNTs/CFME

**Figure 9:**
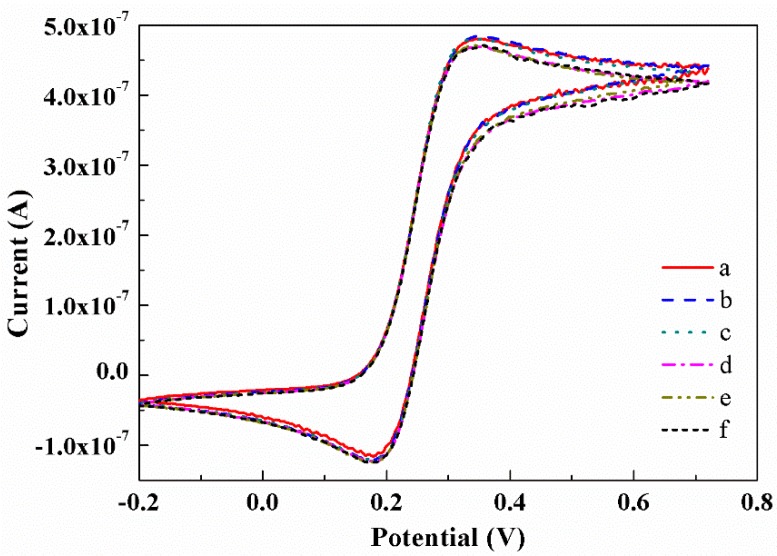
Cyclic voltammograms of the CNTs/CFME in a 5.0 mM K_4_Fe(CN)_6_ (in 1.0 M KCl) at a scan rate of 0.01 V·s^−1^. (**a**) the first; (**b**) the tenth; (**c**) the twentieth; (**d**) the first after 104 days; (**e**) the tenth after 104 days; (**f**) the twentieth after 104 days.

The reproducibility of the CNTs/CFME-T15—with the largest CNT content and the best reflection of the stability of CNTs/CFMEs—was tested by consecutive cyclic potential scans in the 5.0 mM K_4_Fe(CN)_6_ (in 1.0 M KCl), at a scan rate of 0.01 V·s^−1^. After 20 consecutive cycles, the CNTs/CFME-T15 was removed from the solution, rinsed in deionized water, and exposed to the air for about 104 days. Subsequently, the same cyclic voltammetry test was performed again. The first, tenth, and twentieth CV curves before and after 104 days are shown in Figure 9. The results indicate that all of the CV curves are nearly identical with constant response currents, indicating that the CNTs/CFME is exceptionally stable and has extraordinary reproducibility. This is largely attributed to the CNT being a remarkable electrode material that possesses high stability and outstanding reproducibility [37].

## 4. Conclusions

We fabricated a series of carbon fiber microelectrodes modified with carbon nanotubes (CNTs/CFMEs) using different process parameters. The effective working electrode of a CNTs/CFME was made by Ni electroless plating, followed by CVD processes. The encapsulation of the CNTs/CFME was done via a simple process using an insulated copper wire and epoxy resin sealing. The as-prepared CNTs/CFMEs displayed quasi-reversible electrode behaviors in cyclic voltammetry and a quasi-steady state in chronoamperometry. CNTs are validated to be able to significantly enhance the electrical conductivity of the CFs. Compared with a bare CFME, a CNTs/CFME shows a better CV curve, exhibiting a higher distinguishable redox peak and response current. The modified CNTs can provide the CNTs/CFME with a large effective electrode area, substantially increasing the active sites and current density. Consequently, a CNTs/CFME exhibits better electrochemical properties in comparison with the unmodified CFME, in terms of its remarkable reversibility, electrocatalytic activity, and high mass transfer rate. Moreover, a CNTs/CFME is shown to have extraordinary stability and reproducibility, making it more applicable as a sensor in the domains of microscopic biochemical analyses and measurements.

## Figures and Tables

**Table 1:** Formulations of the Ni electroless plating bath.

Chemical	Formula	Amount (g·L^−1^)
Nickel sulfate	NiSO_4_·6H_2_O	40
Sodium hypophosphite	NaH_2_PO_2_·H_2_O	20
Sodium citrate	NaC_6_H_5_O_7_·2H_2_O	100
Ammonium chloride	NH_4_Cl	50
Ammonium hydroxide	NH_3_·H_2_O	pH adjustment

Noted: electroless plating was conducted at 75 °C, pH 8 and for 2 min.

**Table 2:** The linear fitting equations of the *i_qss_* versus 1/ln*τ*.

Microelectrode	The Fitted Linear Regression Equations	Coefficient of Determination
CFME-T0	*i_qss_* = 4.39523 × 10^−7^ 1/ln*τ* − 4.39466 × 10^−8^	0.99678
CNTs/CFME-T5	*i_qss_* = 5.49772 × 10^−7^ 1/ln*τ* + 1.47824 × 10^−8^	0.9974
CNTs/CFME-T10	*i_qss_* = 1.11411 × 10^−6^ 1/ln*τ* + 5.64842 × 10^−9^	0.99049
CNTs/CFME-T15	*i_qss_* = 1.3068 × 10^−6^ 1/lnτ + 6.75016 × 10^−8^	0.99684

**Table 3:** The effective surface area and quasi-steady state current density of the as-prepared microelectrodes.

Microelectrode	Effective Surface Area (cm^2^)	Quasi-Steady State Current Density (A/cm^2^)
CFME-T0	2.39036 × 10^−5^	0.751 × 10^−3^
CNTs/CFME-T5	4.27135 × 10^−5^	2.19 × 10^−3^
CNTs/CFME-T10	12.9838 × 10^−5^	1.47 × 10^−3^
CNTs/CFME-T15	20.3059 × 10^−5^	1.48 × 10^−3^
